# Outcomes of COVID-19 Critically Ill Extremely Elderly Patients: Analysis of a Large, National, Observational Cohort

**DOI:** 10.3390/jcm11061544

**Published:** 2022-03-11

**Authors:** Stefan Andrei, Liana Valeanu, Mihai Gabriel Stefan, Dan Longrois, Mihai Popescu, Gabriel Stefan, Cosmin Balan, Raed Arafat, Dan Corneci, Gabriela Droc, Serban-Ion Bubenek-Turconi, on behalf of the COVATI-RO Collaborative

**Affiliations:** 1 Department of Anaesthesiology and Intensive Care, Carol Davila University of Medicine and Pharmacy, 8 Eroii Sanitari Blvd., 050474 Bucharest, Romania; mihai.popescu@umfcd.ro (M.P.); dan.corneci@gmail.com (D.C.); gabidroc@gmail.com (G.D.); bubenek@alsys.ro (S.-I.B.-T.); 2 Cardiac Anaesthesiology and Intensive Care Department I, Prof. Dr. C. C. Iliescu Emergency Institute for Cardiovascular Diseases, 258 Fundeni Road, 022328 Bucharest, Romania; liana.valeanu@yahoo.com (L.V.); cosmin13mara@yahoo.com (C.B.); 3 Cardiac Anaesthesiology and Intensive Care Department II, Prof. Dr. C. C. Iliescu Emergency Institute for Cardiovascular Diseases, 258 Fundeni Road, 022328 Bucharest, Romania; mihaisteph@yahoo.com; 4 Department of Anaesthesiology and Intensive Care, Bichat-Claude Bernard University Hospital, Sorbonne Universités, INSERM UMR 1148, 46 Rue Henri Huchard, 75018 Paris, France; dan.longrois@aphp.fr; 5 Anaesthesiology and Intensive Care Department III, Fundeni Clinical Institute, 258 Fundeni Road, 022328 Bucharest, Romania; 6 Nephrology Department, Carol Davila University of Medicine and Pharmacy, 8 Eroii Sanitari Blvd., 050474 Bucharest, Romania; gabriel_stefan@rocketmail.com; 7 Department for Emergency Situations, Ministry of Internal Affairs, 1 Revolution Sq., 030167 Bucharest, Romania; arafatr@smurd.ro; 8 Anaesthesiology and Intensive Care Department I, Central Military University Emergency Hospital, 134 Plevnei Road, 010825 Bucharest, Romania; 9 Anaesthesiology and Intensive Care Department I, Fundeni Clinical Institute, 258 Fundeni Road, 022328 Bucharest, Romania

**Keywords:** elderly, ICU, mechanical ventilation, mortality, outcomes, COVID-19

## Abstract

Background. During the COVID-19 pandemic, resource allocation became a major problem in globally overwhelmed ICUs. The main goal of this study was to describe the clinical characteristics of the very elderly patients (aged ≥ 80 years) with COVID-19 admitted in Romanian ICUs. The study objectives were to evaluate and determine the factors associated with ICU mortality. Methods. We designed a national, multicentric, observational platform with prospective enrolment. This study included patients aged ≥ 80 years admitted in Romanian ICUs with SARS-CoV-2 infection from March 2020 to December 2021. Results. We included 1666 patients with a median age of 83 years and 78% ICU mortality. Male sex, dyspnoea, lower Glasgow Coma Scale and lower SpO2 at ICU admission, the need for mechanical ventilation (MV), and corticosteroid use were independently associated with mortality. A total of 886/1666 (53%) elderly patients underwent invasive mechanical ventilation, with a mortality of 97%. The age impact on mortality was confirmed by a 1:1 propensity matching with less elderly ICU patients. Conclusion. In extremely elderly patients with COVID-19 admitted in the ICU, mortality is high, particularly when requiring MV. Therapy should be directed towards the optimization of less invasive ventilatory methods and the use of MV and corticosteroids only in highly selected patients.

## 1. Introduction

The allocation of intensive care unit (ICU) beds for elderly patients has always been a matter of concern. Older age is inversely associated with ICU survival and long-term survival after ICU discharge [1,2]. In terms of quality of life, elderly patients discharged from the ICU show worse outcomes compared to younger survivors or age-matched non-hospitalised controls [3]. Various studies have already addressed age-related mortality in large cohorts of critically ill patients before the SARS-CoV-2 (COVID-19) pandemic [4,5].

During the surges of the COVID-19 pandemic, scarce ICU resource allocation became a major problem for decision makers and health care workers in a globally overwhelmed ICU [6]. Different published cohorts worldwide identified age as an independent risk factor for disease severity and hospitalisation, with increased rates of poor outcome and mortality amongst senior patients [7,8,9,10,11]. The burden of COVID-19 seems much heavier on older patients than on younger adults and children [12,13]. The allocation of ICU beds to elderly patients was not only costly and associated with poor outcomes, but would also possibly withhold valuable ICU resources from younger patients. Consequently, the problem of ICU beds allocation to elderly patients during the COVID-19 pandemic has been addressed heterogeneously by different health care systems [14,15,16], and this challenging age category was targeted by different public health measures [13].

To relieve the pressure on healthcare units, some stakeholders adopted different strategies for ventilator and ICU bed allocation based on age, comorbidities, or severity scores [14,15,16]. Some elderly patients chose not to be mechanically ventilated, through anticipated directives [17]. Furthermore, the limitation of life-sustaining treatments, such as the withholding or withdrawal of active treatment, was more frequent in older and more severe patients [18].

In Romania, due to local ethical and cultural considerations, the limitation of medical treatment for in-hospital patients was not the object of structured decisions and therefore, no additional triage criteria for ICU admission were considered by the Romanian authorities during the pandemic. Age was not considered a discriminating factor between patients with proven COVID-19 infection who met ICU admission criteria, regardless of the availability of ICU beds nationwide.

There are already some small reported cohorts and trials on the particularities of treatment and outcomes of COVID-19 patients older than 80 years [19,20,21]. However, more epidemiological data are needed to better understand the prognosis and management of these patients. The possibility exists that information on elderly patients admitted to the ICU from different health care systems during the COVID-19 epidemic would allow the international and national communities to improve the decision-making process for ICU bed allocation, even after the COVID-19 pandemic.

The main goal of this study was to describe the clinical characteristics of the elderly patients (aged ≥ 80 years) with COVID-19 admitted in Romanian ICUs. The study objectives were to evaluate ICU mortality, to determine the factors associated with ICU mortality, and to determine whether age per se is associated with ICU mortality.

## 2. Materials and Methods

### 2.1. Study Design, Setting, and Participants

This study included patients with age ≥ 80 years admitted in Romanian ICUs with SARS-CoV-2 infection. All patients hospitalised from March 2020 to December 2021 were included. The patients were followed until ICU death or ICU discharge. The patients without confirmed ICU outcomes (discharge or death) were excluded from data analyses.

The data collection had a national, multicentric, observational design with prospective enrolment.

### 2.2. Variables and Definitions

We collected data on the following variables: age, gender, prior co-morbidities, symptoms at ICU admission, clinical presentation, specific therapies during the ICU stay (mechanical ventilation and respiratory therapies, extra-corporeal therapies, pharmacological therapies), clinical outcomes (mechanical ventilation duration, hospital stay, ICU stay, mortality, SOFA score), and complications.

The definitions and diagnostic criteria for acute respiratory distress syndrome (ARDS), acute kidney injury (AKI), major adverse cardiac events (MACE), and myocardial infarction are provided in Supplemental Table S1.

### 2.3. Data Sources and Management

Data were collected anonymously using a national online platform, designed and financed by the Romanian Society of Anaesthesia and Intensive Care. The platform was named COVATI-RO. All the ICUs across the country treating critically ill patients with COVID-19 were asked to participate. The data were collected by the treating intensivists at ICU admission and at the point of clinical worsening requiring endotracheal intubation or prone position ventilation. The file for each patient was closed at the moment of death or discharge from the ICU.

The online platform was conceived in March 2020, at the beginning of the pandemic in Romania. General characteristics (age, gender), past medical history, presenting symptoms and clinical status, ICU complications and evolution, treatment, and ICU survival were noted.

### 2.4. Study Size and Statistical Analyses

No formal sample size analysis was calculated a priori, given the unpredictable evolution of the pandemic at the moment of the conception of the prospective platform.

Continuous variables are expressed as median with (25–75%) interquartile range (IQR). Categorical variables are expressed as numbers (percentages). Normal distribution for continuous variables was evaluated through histograms and the Shapiro–Wilk test. The Student’s *t*-test or Mann–Whitney U test were used, as appropriate, for comparisons of continuous variables. The chi-square or Fisher’s test were used to compare categorical variables, as appropriate.

The factors associated with ICU mortality were first determined using univariate logistical regression, followed by a multivariate regression model. The conditions of validity for the multivariate regression model were verified to have at least 10 events for each variable in the model. As some variables were characterizing the same or similar organ dysfunction, a principal component analysis was complementarily performed, considering the factors associated with ICU mortality in univariate analyses.

To determine the impact of age on ICU mortality and complications, we performed a propensity score matching using a nearest-neighbour algorithm with 1:1 matching without replacement and a calliper distance of less than 0.01. The elderly patients (≥80 years) were matched with immediate less-elderly patients (65 ≤ age < 80 years). The covariates of clinical severity at ICU admission, associated comorbidities, and ICU treatment and management were considered in the matching algorithm. The adequacy of covariate balance in the matched sample was assessed using absolute standardised mean differences. Quantitative and qualitative paired variables after propensity matching were compared by using the Wilcoxon matched pair test or the McNemar test, as appropriate.

A principal component analysis and a factorial analysis were performed using the variables independently associated with mortality in multivariate analyses. 

The missing data are presented. No missing data imputation strategy was used for this study. 

Data were analysed using SPSS^®^ (IBM SPSS Statistics for Windows, Version 21.0., IBM Corp, Armonk, NY, USA) and using RStudio (Version 1.1.447—© 2009–2018 RStudio, Inc., Boston, MA, USA). The threshold for statistical significance was set to *p* < 0.05. 

The R packages “MatchIt” and “Psy” were used for the propensity score matching and factorial analysis.

### 2.5. Ethical Aspects

The ethical approval for this study was granted by the Department of Medical Education and Research of the Romanian Society of Anaesthesia and Intensive Care (decision no.5/07.04.2020). The patients’ data were completely anonymised, and the analysis was performed blindly as to patients’ identity and to the centre of inclusion.

The manuscript was drafted according to the STROBE guidelines. The STROBE checklist is available as a supplementary file to this manuscript.

## 3. Results

### 3.1. Patients’ Characteristics

**Figure 1:**
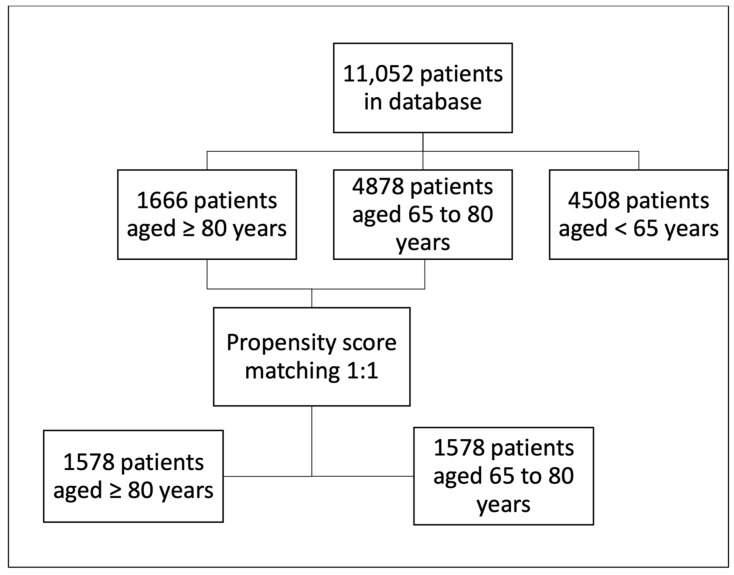
Study flowchart.

From the 11,052 patients enrolled in the COVATI-RO dataset, 15% (1666/11052) met the inclusion criteria of the present study (Figure 1).

The studied population included 1666 patients, with a median age of 83 years and equal sex ratio. As expected, the comorbidities burden was high: arterial hypertension (80%) was the most frequent, followed by ischemic heart disease (58%), heart failure (46%), diabetes mellitus type 2 (31%), and chronic kidney disease (27%) (Table 1).

### 3.2. ICU Admission and Management

The most common symptoms at ICU admission were dyspnoea, cough, and fever; the median oxygen saturation was 84%, and more than a third of the patients had been diagnosed with ARDS (Table 1).

The median maximum Sequential Organ Failure Assessment (SOFA) score was 8 and the median ICU length of stay was 5 days (Table 2).

Overall, non-invasive ventilation (NIV) was used for 729 (44%) patients, high-flow oxygen (HFO2) for 698 (42%) patients, and MV was performed (initially or following other modes) for 886 (54%) patients.

A total of 225/1666 (14%) patients were managed with HFO2 only, 201 (12%) with NIV only, and 113 (6%) with a combination of NIV and HFO2; 325 (19.5%) patients directly underwent invasive MV. A total of 146 (9%) patients were treated with HFO2 and MV, 201 (12%) patients had NIV and MV, and 214 (13%) patients had HFO2, NIV, and MV combined.

A neuromuscular blockade was used for 10% of the intubated patients for a median duration of 2 days. The median highest positive end-expiratory pressure (PEEP) used was 10 cm H_2_O, which was similar in both survivors and non-survivors. Only 11% of the patients were treated using prone position ventilation, with a median number of sessions of 4. No patient underwent extracorporeal membrane oxygenation (ECMO) support in our reported cohort.

The most administered drugs for COVID-19 were steroids, followed by lopinavir/ritonavir, hydroxychloroquine, remdesivir, and tocilizumab (Table 2). Overall, 1295 patients (78%) received corticosteroids, dexamethasone being preferred in over 80% of the cases.

### 3.3. ICU Complications

The median length of stay was of 5 (2–10) days for all cohort, longer for survivors than for non-survivors, 6 (4–11) vs. 5 (2–9), *p* < 0.001, respectively.

During the ICU stay, 385/1666 (23%) patients developed a new sepsis episode, 224 (14%) patients experienced at least one major cardiovascular event, and 85 (5%) exhibited stroke; 456 (27%) patients developed AKI, of whom 36% (163/456) had stage II AKI and 29% (132/456) had stage III AKI, according to AKIN classification. It is noted that 10% (47/456) of the patients with AKI received continuous renal replacement therapy (CRRT). A total of 218 patients (13%) required vasopressor support for hemodynamic instability during their ICU stay.

### 3.4. ICU Mortality and Predictors

The overall mortality in this cohort of elderly patients requiring ICU admission due to COVID-19 was 78%. Patients who died were more frequently males, more often presented with dyspnoea and cough, and had significantly lower oxygen saturation upon ICU admission. Moreover, they had a higher median SOFA score, a significantly lower Glasgow Coma Scale, and presented with almost twice as many cases of ARDS at admission (Table 1 and Table 2). We found no relationship between mortality and the recorded comorbidities (Table 1).

The mortality was extremely high in the group of patients who needed mechanical ventilation during the ICU stay, reaching 97%, with only 29/886 patients having been successfully extubated.

Systemic corticosteroids were used significantly more often and there was a trend toward a higher use of tocilizumab in the non-survivors’ group (Table 2).

In the multivariate logistic regression model, male sex, dyspnoea upon ICU admission, decreased value of GCS, and lower oxygen saturation at ICU admission, the need for mechanical ventilation, and the use of corticosteroids were independently associated with mortality (Table 3). The use of HFO2 only or in combination with NIV was associated with survival (Table 3). Several sensitivity analyses considering different combinations of the categories of ventilatory support, or excluding the respiratory management, showed similar results (using the same independent predictors). The principal component analyses for potential interactions between these predictors are shown in Supplemental Figures S1–S3 and Supplemental Table S2.

### 3.5. Comparison with Less-Elderly Patients (65 ≤ Age < 80 Years) after Propensity Matching

The study patients were 1:1 matched with less elderly ICU patients from the same national database hospitalised during the same time span (Figure 1). The patients’ characteristics before and after propensity matching are presented in Supplemental Table S3. The adequacy of the covariate balance was satisfactory (Supplemental Figure S4).

As expected, the older patients had a higher mortality (77.9% vs. 72.9%, *p* < 0.001), with a similar ICU length of stay.

### 3.6. The Differential Role of COVID-19 Surges on Corticotherapy Effect

Considering the daily new cases of COVID-19, based on the official database of the Romanian Government [22,23], we arbitrarily classified three periods of surge: surge A (from March 2020 to January 2021, 1162 (70%) patients), surge B (from February 2021 to June 2021, 374 (23%) patients), and surge C (from July 2021 to December 2021, 130 (8%) patients).

The corticosteroids administration was associated with ICU mortality during periods A (OR 1.8 (1.34, 2.42, *p* < 0.001) and B (OR 2.24 (1.19, 4.21), *p* = 0.013), but not period C (OR 1.33 (0.27, 6.71), *p* = 0.727).

### 3.7. Evaluation of Interaction Effect between Corticosteroids Administration and Gender and Comorbidities

No interaction effect on mortality between sex and corticosteroids administration was found in the logistic regression model (*p* = 0.692). No statistically significant interaction effect of excess mortality was observed between corticosteroids administration and each collected comorbidity. 

## 4. Discussion

### 4.1. Patients’ Characteristics and ICU Mortality

In the present study, we report data from one of the largest cohorts of extremely elderly ICU patients. As initially hypothesised, the local context of a lack of age-related ICU admission restrictions allowed for the inclusion of more patients than usually reported for this age category. The percentage of patients > 80 years old (15%) indicates an over-representation of this age group, as the percentage of citizens > 80 years in Romania at the last validated census was only 3.6% [24]. Unsurprisingly, we found a very high mortality, particularly for the patients receiving invasive mechanical ventilation. For the same age category, the overall mortality was lower than that reported by a smaller regional Turkish cohort (80.5%) [19], but slightly higher than in most Western European published studies: 55% in an Italian cohort [9], 62.5% and 66.8% in French cohorts [25,26], and 72% in a German study [27]. The COVIP study observed a mortality at 30 days (not general in-hospital mortality) of 41% in patients aged 70 or older, which increased to 48% at 90 days, and was 67% in frail patients [28]. Raw comparisons are difficult to make, as different age cut-offs are used in COVID-19-related articles: 60-years, 65-years, 70-years, 75-years, 80-years, and 85-years [4,10,19,21,25,28,29,30,31].

In our cohort, 886 (53%) elderly patients underwent MV, either from the beginning of ICU admission or following a less invasive ventilator support, and their mortality was extremely high (97%). Some North American cohorts previously reported a fatality rate of 90% in patients older than 80 years on MV [32,33]. Other published cohorts usually reported lower, but still high, mortality [34]. Some cohorts have reported an inverse association between age and MV in COVID-19 patients, suggesting that there is an inherent bias towards less invasive treatment in these patients, regardless of the existence of clear limitation of treatment orders or not [33]. In our cohort, univariate analysis with invasive MV as the dependent variable did not show a significant association with age (OR 0.9, 95% CI 0.98–1.04). This suggests that there was no inherent bias towards less intubation in patients older than 80 years.

The poor outcomes of the mechanically ventilated elderly, in terms of mortality and long-term life quality, open the discussion about “a bad death” and about the benefits of such an invasive ICU approach [18,35]. However, the ethical controversy regarding ICU admission for the elderly is ongoing, as age alone might not be an appropriate predictor for hospital outcomes [36,37]. The assessment of individual benefit-risk balance and the degree of frailty, rather than age or comorbidities alone, are better predictors of COVID-19 outcomes [28,38].

Our results should be viewed cautiously, as some therapies, such as prone position ventilation, were not implemented as frequently as reported by other studies. Only 22 patients in the survivor group (75.8% of those with invasive MV in the survival group) and 153 patients in the non-survivor group (17.8% of invasively ventilated patients in the non-survivor group) were treated using prone ventilation.

Interestingly, contrary to the results in younger patients, the comorbidities were not independent predictors of mortality in the elderly, confirming the results of a smaller study [19]. A possible explanation is the relatively high whole cohort incidence of some co-morbidities, such as arterial hypertension and ischemic heart disease, as well as the low incidence of some others, such as autoimmune disease. The reported comorbidities for elderly patients in this study were also identified in previous publications [39]. There are slight differences regarding the prevalence of arterial hypertension and ischemic heart disease, which seem higher in our cohort. This may be partially explained by differences in ethnicity and risk factors, but also by less efficient screening and prevention strategies.

The lower impact of comorbidities on mortality, confirmed by the propensity matching analysis, is in favour of a very strong role of age as a predictor of survival. The elderly patients have a degree of immunosuppression, due to immunosenescence (38). However, we did not analyse geriatrics-specific scores and indices of frailty, which are known to have a better sensitivity for worse hospital outcomes in the elderly [18,28,36,38,40].

### 4.2. Corticosteroid Prescription in Elderly Patients

We found a higher use of corticosteroids in the non-survivor group (80% vs. 68%, *p* < 0.001). In this cohort, corticosteroid prescription was independently associated with mortality in the multivariate analysis (Table 3). There are some previously published data suggesting similar results, as a secondary analysis of the COVIP study having found a higher 30-day-mortality in critically ill COVID-19 patients, aged 70 years or older, who received steroids as part of their treatment [41]. However, there is no recommendation yet for the differentiated treatment of elderly patients. Other previous studies, such as RECOVERY, also did not find a beneficial effect of corticosteroid use in patients aged 70 or older [42]. We found that the impact of corticosteroids on mortality differs according to the period of COVID-19 surge, but we did not analyse the SARS-CoV-2 variants. A few researchers have already suggested that the natural history of the different SARS-CoV-2 variant diseases responded differently to corticosteroids [41].

There have been publications addressing the different immune profiles of males and females, which could impact the clinical response to a variety of molecules, including corticosteroids [43]. In our cohort, no interaction effect was found between gender and corticosteroids administration and mortality.

Further research into the benefit of corticosteroids in the elderly should be pursued, as the lack of benefit, or even harm, of this therapy in this subgroup could lead to individualised, tailored therapies for these patients.

### 4.3. Tocilizumab Administration in Elderly Patients

Tocilizumab was not associated with a decreased mortality in this cohort in the univariate analysis, and it was not included in the multivariate regression model. However, there was a low use of this drug; it was used in only 8% (138) of patients. The use of tocilizumab in this age group has rarely been addressed; similarly, in other cohorts, we found a low use of this high-risk therapy [19]. In the RECOVERY trial, there were 472 patients 80 or older, with a risk ratio for mortality of 0.92, 95% (CI 0.73–1.15), therefore suggesting a reduction in tocilizumab benefit as compared to that for younger patients [42]. As there is very little adequate quality data regarding this drug in elderly patients, clinicians should prescribe tocilizumab on an individualised basis. Further research via randomised controlled studies should be undertaken.

### 4.4. Elderly Definition Debate

The elderly patient has been previously defined as a patient older than 65 years [1]. However, recent literature has shifted towards analysis of extreme age, defining the very old patient as more than 75 to 85 years, in various cohorts. In our cohort, we defined the very old patient as 80 years or older, as octogenarians have been included in multiple recent prospective and retrospective designs, both in anaesthesia care and in ICU studies [44,45]. Age thresholds, combined or as an alternate to clinical scores and the presence of co-morbidities, have been used for triage at ICU admission in situations of limited resources [14]. Moreover, considering the life expectancy at birth in Romania (75.3 years in 2018), our study cohort should rather be categorised as extremely elderly [46].

### 4.5. The Clinical Relevance of Our Results

First, our findings confirmed the high mortality in extremely elderly patients requiring invasive MV. Because there was no effect of age on the probability to be mechanically ventilated, we consider that our results are valuable and are able to estimate a non-biased effect of MV on the risk of death in elderly patients. In this study, only ICU mortality was collected from the follow up, but 30-days and 1-year mortality are expected to be even higher. Also, the post-ICU complications and the quality of life were not analysed, and we were not able to specifically collect indices of frailty. However, our results do not favour a very invasive ICU management in this age category. We consider that efforts should be directed towards the optimization of less invasive ventilatory methods and the use of MV only in highly selected patients.

Second, mortality was significantly higher in elderly patients receiving corticosteroids and corticotherapy was independently associated with increased mortality. These results are similar to previously reported data questioning the routine use of corticosteroid treatment in elderly COVID-19 patients [41]. Even though this study was not specifically designed to evaluate the effects of corticosteroids in elderly patients, the results suggest that this treatment might not be as beneficial as expected from the results in younger patients. The principal component analyses suggested a possible interaction between the presence of dyspnoea at admission and the corticosteroids effect. Finally, the observational design of this study does not allow any causality inference. Further randomised trials are necessary to elucidate this question.

### 4.6. Limitations

First, the generalisability of our findings should be viewed cautiously, as the clinical management, although standardised through national guidelines based on international recommendations, might have been different from the Western/other countries’ standards. One example is the infrequent use of prone ventilation in our cohort. Furthermore, we did not collect information about important ventilatory settings such as the plateau pressure.

Second, not all associated comorbidities were noted. One notable absence is the ponderal status, as obesity is a known factor associated with respiratory dysfunction, and malnutrition is associated with worse overall outcomes in the elderly. The APACHE II score on ICU admission was not collected, the SOFA score having been chosen instead. We also need to point out that some patients might have had a suboptimal medical care before the pandemic and incomplete or inaccurate health records, and some comorbidities could have been underdiagnosed/undertreated [47].

Third, we did not collect and analyse frailty characteristics and scores in this study. Different frailty scores are known to be better predictors of outcomes in the geriatric population [38,40].

Fourth, we did not collect and analyse laboratory data. The elderly adults are known to have a high burden of comorbidities that were not completely characterised in our study. Moreover, some laboratory data are markers of circulatory failure (lactate, natriuretic peptides concentrations) or respiratory failure (PaO_2_/FiO_2_ ratio). Although some dysfunctions and complications were collected in a qualitative manner, the quantitative approach might have been more discriminative. Fifth, we did not note and analyse some important prophylaxis and treatments, such as anticoagulation or the administration of vaccines.

## 5. Conclusions

In this large cohort of very elderly patients from an Eastern European country, ICU mortality was high and was independently predicted by dyspnoea, low SpO2 and GCS score values at admission, and by the need for invasive MV and corticosteroid administration during ICU stay.

In extremely elderly patients with COVID-19 admitted in ICU, therapy should be directed towards the optimization of less invasive ventilatory methods and the use of MV and corticosteroids only in highly selected patients.

Corticosteroids remain of controversial benefit in the elderly population and should be further addressed in controlled studies.

## Figures and Tables

**Table 1:** Patients’ characteristics at ICU admission.

	All Cohort n = 1666	Survivors n = 361	Non-Survivors n = 1305	*p*-Value	Missing n (%)
Age (years), median	83 [81–86]	83 [81–86]	83 [81–86]	0.1	
Male sex, n (%)	834 (50)	157 (44)	677 (52)	0.005	
Clinical Status on ICU Admission					
Shiver (yes), n (%)	239 (14)	52 (14)	187 (14)	0.9	
Headache (yes), n (%)	177 (11)	43 (12)	134 (10)	0.3	
Myalgia (yes), n (%)	266 (16)	63 (18)	203 (16)	0.3	
Fever (yes), n (%)	561 (34)	100 (28)	461 (35)	<0.01	
Nausea (yes), n (%)	88 (5)	22 (6)	66 (5)	0.4	
Cough (yes), n (%)	876 (53)	180 (50)	696 (53)	0.2	
Diarrhoea (yes), n (%)	79 (5)	18 (5)	61 (5)	0.8	
Dyspnoea (yes), n (%)	1288 (77)	247 (68)	1041 (80)	<0.001	
SpO2 (%), median	84 [70–91]	93 [87–97]	80 [70–90]	<0.001	
ARDS (yes), n (%)	595 (36)	89 (25)	506 (39)	<0.001	
GCS, median	12 [6–15]	15 [13–15]	12 [4–15]	<0.001	
SOFA	6 [4–12]	5 [3–8]	7 [4–13]	<0.001	262 (15.7)
Associated Medical History					
Ischemic heart disease (yes), n (%)	972 (58)	204 (57)	768 (59)	0.4	
Autoimmune disease (yes), n (%)	20 (1)	3 (1)	17 (1)	0.4	
Dialysis patient (yes), n (%)	43 (3)	14 (4)	29 (2)	0.07	
COPD (yes), n (%)	156 (9)	42 (12)	114 (9)	0.09	
Past or current cancer (yes), n (%)	151 (9)	32 (9)	119 (9)	0.8	
Chronic kidney disease (yes), n (%)	448 (27)	89 (25)	359 (28)	0.2	
Diabetes type 1 (yes), n (%)	18 (1)	3 (1)	15 (1)	0.6	
Diabetes type 2 (yes), n (%)	509 (31)	105 (29)	404 (31)	0.4	
Heart failure (yes), n (%)	764 (46)	171 (47)	593 (45)	0.5	
Arterial hypertension (yes), n (%)	1339 (80)	294 (81)	1045 (80)	0.5	

ARDS—acute respiratory distress syndrome; COPD—chronic obstructive pulmonary disease; GCS—Glasgow coma scale; ICU—intensive care unit; SOFA—sequential organ failure assessment.

**Table 2:** Patients’ management in ICU.

Variables	All Cohort n = 1666	Survivors n = 361	Non-Survivors n = 1305	*p*-Value	Missing n (%)
Maximum SOFA, median	8 [4–14]	5 [3–8]	9 [5–15]	<0.001	
Respiratory management					
Non-invasive respiratory management					
HFO2 (yes), n (%)	698 (42)	194 (54)	504 (39)	<0.001	
HFO2 only (yes), n (%)	225 (14)	125 (35)	100 (8)	<0.001	
NIV (yes), n (%)	729 (44)	106 (29)	623 (48)	<0.001	
NIV only (yes), n (%)	201 (12)	39 (11)	162 (12)	0.406	
HFO2 and NIV (yes), n (%)	113 (7)	60 (17)	53 (4)	<0.001	
Invasive respiratory management					
Mechanical ventilation (yes), n (%)	886 (53)	29 (8)	857 (66)	<0.001	
MV only (yes), n (%)	325 (20)	15 (4)	310 (24)	<0.001	
HFO2 and MV (yes), n (%)	146 (9)	7 (2)	139 (11)	<0.001	
NIV and MV (yes), n (%)	201 (12)	5 (1.5)	196 (15)	<0.001	
HFO2, NIV, and MV (yes), n (%)	214 (13)	2 (1)	212 (16)	<0.001	
Neuromuscular blockade (yes), n (%)	162 (10)	5 (1)	157 (12)	<0.001	
Maximum PEEP (cm H_2_O), median	10 [8–12]	8 [7–10]	10 [8–12]	0.2	
Prone ventilation (yes), n (%)	175 (11)	22 (6)	153 (12)	<0.01	
Treatment					
Corticosteroids (yes), n (%)	1295 (78)	246 (68)	1049 (80)	<0.001	
Remdesivir (yes), n (%)	316 (19)	48 (13)	268 (21)	<0.01	
Hydroxychloroquine (yes), n (%)	356 (21)	84 (23)	272 (21)	0.3	
Lopinavir/Ritonavir (yes), n (%)	385 (23)	91 (25)	294 (23)	0.2	
Tocilizumab (yes), n (%)	138 (8)	22 (6)	116 (9)	0.08	

HFO2—high flow oxygen; ICU—intensive care unit; MV—mechanical ventilation; NIV—non-invasive ventilation; PEEP—positive end-expiratory pressure; SOFA—sequential organ failure assessment.

**Table 3:** The univariate and multivariate logistic regression analysis for ICU death as a dependent variable.

Variables	Univariate		Multivariate	
	OR (95% CI)	*p*-Value	OR (95% CI)	*p*-Value
Age (per 1 year), reference 80 years	1.02 (0.99, 1.05)	0.1	-	-
Gender (male), reference female	1.40 (1.10, 1.77)	0.005	1.36 (1.00, 1.85)	0.049
Past medical history				
Ischemic Heart Disease (yes)	1.10 (0.87, 1.39)	0.4	-	-
Cancer (yes)	1.03 (0.68, 1.55)	0.8	-	-
Chronic Kidney Disease (yes)	1.16 (0.88, 1.51)	0.2	-	-
Diabetes Type 2 (yes)	1.09 (0.84, 1.41)	0.4	-	-
Heart Failure (yes)	0.92 (0.73, 1.16)	0.5	-	-
HTA (yes)	0.91 (0.68, 1.23)	0.5	-	-
Symptoms on admission				
Dyspnoea (yes)	1.82 (1.40, 2.36)	<0.001	1.81 (1.27, 2.59)	0.001
Fever (yes)	1.43 (1.1, 1.84)	0.007	1.24 (0.89, 1.73)	0.205
SpO2 < 90% on admission (yes)	5.30 (4.13, 6.8)	<0.001	3.78 (2.76, 5.16)	<0.001
ARDS (yes)	1.93 (1.48, 2.52)	<0.001	1.07 (0.76, 1.51)	0.7
GCS on admission (each scale point)	0.75 (0.72, 0.79)	<0.001	0.81 (0.76, 0.86)	<0.001
Evolution in ICU				
SOFA (each score point)	1.13 (1.10, 1.16)	<0.001	1.03 (1.01, 1.07)	0.017
HFO2 only (yes) *	0.16 (0.12, 0.21)	<0.001	0.37 (0.27, 0.52)	<0.001
HFO2 + NIV only (yes) *	0.21 (0.14, 0.31)	<0.001		
HFO2 (yes)	0.54 (0.43, 0.69)	<0.001	-	
NIV (yes)	2.19 (1.70, 2.82)	<0.001	-	-
NIV only (yes)	1.17 (0.81, 1.7)	0.406	-	
Mechanical ventilation (yes)	21.90 (14.73, 32.55)	<0.001	6.78 (4.27, 10.77)	<0.001
Mechanical ventilation only (yes)	7.2 (4.22, 12.24)	<0.001	-	
HFO2 and MV (yes), n (%)	6.03 (2.8, 13)	<0.001	-	
NIV and MV (yes), n (%)	12.58 (5.14, 30.82)	<0.001	-	
HFO2 and NIV and MV (yes), n (%)	34.82 (8.6, 140.84)	<0.001	-	
Neuromuscular blockade (yes)	9.73 (3.96, 23.90)	<0.001	1.93 (0.67, 5.54)	0.222
Prone position (yes)	0.48 (0.30, 0.77)	0.002	0.77 (0.41,1.46)	0.426
Treatment				
Corticosteroids (yes)	1.91 (1.47, 2.48)	<0.001	1.69 (1.18, 2.43)	0.004
Hydroxychloroquine (yes)	0.86 (0.65, 1.14)	0.3	-	-
Lopinavir/Ritonavir (yes)	0.86 (0.65, 1.13)	0.2	-	-
Remdesivir (yes)	1.69 (1.21, 2.35)	0.002	1.28 (0.84, 1.94)	0.252
Tocilizumab (yes)	1.50 (0.93, 2.40)	0.09	-	-

ARDS—acute respiratory distress syndrome; CI—confidence interval; GCS—Glasgow Coma Scale; HFO2—high flow oxygen; HTA—arterial hypertension; MV—mechanical ventilation; NIV—non-invasive ventilation; MACE—major adverse cardiac events; OR—odds ratio; SOFA—sequential organ failure assessment; CRRT—continuous renal replacement therapy; SpO2—pulse oximetry saturation. * In the multivariate analysis, the two categories were treated as one variable.

## Data Availability

Restrictions apply to the availability of these data. The data presented in this study are available on reasonable request from the corresponding author.

## Supplementary Materials

The following supporting information can be downloaded at: https://www.mdpi.com/article/10.3390/jcm11061544/s1, COVATI-RO Collaborative: collaborators and affiliations. Table S1: Definitions [48,49,50]; Table S2: Factorial analysis (2 factors); Table S3: Propensity matching; Figure S1: Principal component analysis considering the factors independently associated with ICU mortality in elderly patients; Figure S2: Spherical representation of principal component analysis considering the factors independently associated with ICU mortality in elderly patients; Figure S3: Eigenvalues for factorial analysis considering the factors independently associated with ICU mortality in elderly patients. Three possible explaining factors are possible; Figure S4: Standardized mean difference (SMD) before and after matching, STROBE checklist.
