# Supplementary material for: Outcomes of COVID-19 Critically Ill Extremely Elderly Patients: Analysis of a Large, National, Observational Cohort
Source: J Clin Med. 2022 Mar 11;11(6):1544. doi: 10.3390/jcm11061544 (PMC8951481; doi:10.3390/jcm11061544)
Supplement: Supplementary file 1 [file jcm-11-01544-s001.zip › jcm-1621213-supplementary.pdf]

COVATI-RO Collaborative
Collaborators, Affiliations

Ioana-Marina Grințescu[1,2], Liliana Mirea[1,2], Raluca Ungureanu[1,2], Cristian Cobilinschi[1,2], Ana-Maria Cotae[1,2], Ecaterina Scărlătescu[2,3], Lavinia Brezeanu[2,4], Ruxandra Tulbure[5], Narcis Valentin Tanase[6], Dan Mihai Lantis[6], Augustin Tudose[6], Cristian Plesa[6], Mădălina Duțu[2,7], Silvius Negoiță[2,7], Emanuel Moisa[2,7], Bianca Morosanu[2,8],Anca Drăgan[8], Cornel Robu[8], Teodora Bute[8], Bogdan Prodan[9], Cornelia Predoi[9], Camelia Călin[10], Raluca Havriliuc[11], Lelia Iliescu[12], Ioana Simion[13], Delia Stanciu[14], Cătălin Macovei[15], Florin-Nicolae Taină[15], Viorica-Daniela Mitescu[15], Tudor Vladoiu[15], Andrei Cuibari[15,] Andra Hagiu[15], Manuela Dan[15], Adrian Stănculea[16], Olimpia Iulia Zoican[17], Letiția Coriu[18], Ana-Maria Munteanu[19], Radu Stoica[20], Oana Dumitrașcu[21], Laura Constantinescu[22], Cristina Petrișor[23], Adrian Stef[24], Daniela Ionescu[25], Lucia Herbel[26], Simu Tudor[27], Bianca Burghelea[28], Alexandra Știrbu[29], Liliana Florea[30], Luminița Galbenu[31], Daniela Prunoiu[32], Adrian Ciocanea[33], Georgel Dahnovici[34], Iurie Rotaru[35], Adriana Dobrescu[36], Aurelia Medan[37], Alexandra-Simona Husac[38], Mihai Soare[39], Carolina Susanu[40], Simona Gălbineanu[41], Adrian Măgureanu[42], Emese Jakab[43], Corin-Constantin Manta[44], Mircea-Eugen Frumosu[45], Marius Novac[46], Mihai Negrea[47], Dragos Garofil[48], Anamaria Hărăbor[49], Corina Manole Palivan[50], Lilian Drăgoi[51], Lucian Horhotă[52], Tölcsér Zsolt[53], Sav Hajnal[54], Maria Suditu[55], Ioana Kovacs[56], Iasmina Salim[57], Alexandrina Caba[58], Liviu Antohi[59], Dan Dăscălescu[60], Andrei Cazacenco[61], Florin Manuel Roșu[62], Gabriel Iosep[63], Gheorghe Dorobăț[64], Mihaela Blaj[65], Sergiu Ungureanu[66], Ionuț Soare[67], Alina Rusu[68], Rodica Cilibia[69], Claudia Iuga[70], Judit Kovacs[71], Mircea Stoian[72], Liviu Ungureanu[73], Gabriela Nacu[74], Călin Tiu[75], Viorel Vereș[76], Anamaria Pal[77], Mihai Sava[78], Dorin Stănescu[79], Petru Martin Deutsch[80], Mirela Tamara Poroșnicu[81], Gordana Boanță[82], Delia Radu[83], Mihaela Dănescu[84], Elena Chircov[85], Angel Bologa[86], Ion Chilinciuc[87], Romuald Sorogaila[88], Georgeta Gugonea[89], Luxița Petrescu[90], Oana Antal[91], Mihaiela Marian[92], Janos Szederjesi[93], , Sanda Copotoiu[93], Ioana Grigoras[94,95], Constantin Bodolea[96], Horatiu Moldovan[97,98], Daniela Filipescu[2,9], Dana Tomescu[2,3], Alexandru-Florin Rogobete[99,100], Dorel Sandesc[99,100]

[1] Anaesthesiology and Intensive Care Department, Emergency Hospital, 8 Floreasca Road, 014461, Bucharest, Romania

[2] Anesthesiology and Intensive Care Department, Carol Davila University of Medicine and Pharmacy, 8 Eroii Sanitari Blvd., 050474, Bucharest, Romania

[3] Anaesthesiology and Intensive Care Department III, Fundeni Clinical Institute, 258 Fundeni Road, 022328, Bucharest, Romania

[4] Anaesthesiology and Intensive Care Department I, Fundeni Clinical Institute, 258 Fundeni Road, 022328, Bucharest, Romania

[5] Anaesthesiology and Intensive Care Department II, Fundeni Clinical Institute, 258 Fundeni Road, 022328, Bucharest, Romania

[6] Anaesthesiology and Intensive Care Department I, Central Military University Emergency Hospital, 134 Plevnei Road, 010825, Bucharest, Romania

[7] Anaesthesiology and Intensive Care Department, Elias Emergency University Hospital, 17 Mărăști Blvd. 11461, Bucharest, Romania

[8] Cardiac Anesthesiology and Intensive Care Department I, Emergency Institute for Cardiovascular Diseases Prof. Dr. C. C. Iliescu, 258 Fundeni Road, 022328, Bucharest, Romania

[9] Cardiac Anesthesiology and Intensive Care Department II, Emergency Institute for Cardiovascular Diseases Prof. Dr. C. C. Iliescu, 258 Fundeni Road, 022328, Bucharest, Romania

[10] Anaesthesiology and Intensive Care Department, Sf Ioan Emergency Hospital, 13 Vitan-Bârzești Road, 042122, Bucharest, Romania

[11] Anaesthesiology and Intensive Care Department, Clinical Hospital Nicolae Malaxa, 12 Vergului Road, 02441, Bucharest, Romania
[12] Anaesthesiology and Intensive Care Department I, Clinical Hospital Colentina, 19-21 Ștefan cel Mare Road, 020125, Bucharest, Romania
[13] Anaesthesiology and Intensive Care Department II, Clinical Hospital Colentina, 19-21 Ștefan cel Mare Road, 020125, Bucharest, Romania
[14] Anaesthesiology and Intensive Care Department, Clinical Hospital of Infectious and Tropical Diseases Victor Babeş, 281 Mihai Bravu Road, 030303, Bucharest, Romania
[15] Anaesthesiology and Intensive Care Department, National Institute for Infectious Diseases Matei-Balş, 1 Dr. Calistrat Grozovici Street, 021105, Bucharest, Romania
[16] Anaesthesiology and Intensive Care Department, Clinical Emergency Hospital of Plastic Surgery, Repairs and Burns, 218 Griviței Road, 010713, Bucharest, Romania
[17] Anaesthesiology and Intensive Care Department, Institute for ENT Functional Surgery and Phonoaudiology Prof. Dr. Dorin Hociotă, 21 Mihail Ciocanu Street, 061344, Bucharest, Romania
[18] Anaesthesiology and Intensive Care Department, Coltea Clinical Hospital, 1 I.C. Brătianu Blvd. 30171, Bucharest, Romania
[19] Anaesthesiology and Intensive Care Department, Foisor Clinical Hospital of Orthopedics, Traumatology and Osteo-Articular Tuberculosis, 35-37 Ferdinad I Blvd. 030167, Bucharest, Romania
[20] Anaesthesiology and Intensive Care Department, National Institute of Pneumology Marius Nasta, 90 Viilor Street, 050152, Bucharest, Romania
[21] Anaesthesiology and Intensive Care Department, University Emergency Hospital, 169 Independenței Blvd, 050098, Bucharest, Romania
[22] Anaesthesiology and Intensive Care Department, Clinical Emergency Hospital St. Pantelimon, 340-342 Pantelimon Road, 077145, Bucharest, Romania
[23] Anaesthesiology and Intensive Care Department, Cluj-Napoca County Clinical Emergency Hospital, 3-5 Clinicilor Street, 400000, Cluj-Napoca, Romania
[24] Anaesthesiology and Intensive Care Department, Nicolae Stăncioiu Heart Institute, 19-21 Moților Road, 400001, Cluj-Napoca, Romania
[25] Anaesthesiology and Intensive Care Department, Regional Institute of Gastroenterology and Hepatology Prof. Dr. Octavian Fodor, 19 Coitorilor Street, Cluj-Napoca, Romania
[26] Anaesthesiology and Intensive Care Department, Clinical Hospital of Infectious Diseases Cluj-Napoca, 23 Iuliu Moldovan Street, 400003, Cluj-Napoca, Romania
[27] Anaesthesiology and Intensive Care Department, Clinical Hospital of Pneumology Leon Daniello, 6 Bogdan Petriceicu Hașdeu Street, 400332, Cluj-Napoca, Romania
[28] Anaesthesiology and Intensive Care Department, Clinical Rehabilitation Hospital Cluj-Napoca, 46-50 Viilor Street, 400066, Cluj-Napoca, Romania
[29] Anaesthesiology and Intensive Care Department, Military Emergency Clinical Hospital Dr. Constantin Papilian, 22 General Traian Moșoiu Street, 400132, Cluj-Napoca, Romania
[30] Anaesthesiology and Intensive Care Department, Dej Municipal Hospital, 14-16 1 Mai Street, 405200, Dej, Romania
[31] Anaesthesiology and Intensive Care Department, Pitești Emergency County Hospital, 36 Spitalului Alley, 110283, Pitești, Romania
[32] Anaesthesiology and Intensive Care Department, Câmpulung Municipal Hospital, 8 Doctor Costea Street, 115100, Câmpulung, Romania
[33] Anaesthesiology and Intensive Care Department, Mioveni City Hospital, 131A Dacia Blvd. 115400, Mioveni, Romania
[34] Anaesthesiology and Intensive Care Department, Bacau Emergency County Hospital, 2-4 Spiru-Haret Street, 600114, Bacău, Romania
[35] Anaesthesiology and Intensive Care Department, Ioan Lascar Comănești City Hospital, Spitalului Street, 605200, Comănești, Romania

[36] Anaesthesiology and Intensive Care Department, Oradea Municipal Hospital, 12 Corneliu Coposu Street, 410469, Oradea, Romania
[37] Anaesthesiology and Intensive Care Department, Bistrița Emergency County Hospital, 43 General Grigore Bălan Blvd, 420094, Bistrița, Romania
[38] Anaesthesiology and Intensive Care Department, Emergency County Hospital Mavromati, 11 Arhimandrit Marchian Street, 710221, Botoșani, Romania
[39] Anaesthesiology and Intensive Care Department I, Brăila Emergency County Hospital, 2 Buzăului Road, 810325, Brăila, Romania
[40] Anaesthesiology and Intensive Care Department II, Brăila Emergency County Hospital, 2 Buzăului Road, 810325, Brăila, Romania
[41] Anaesthesiology and Intensive Care Department, Brașov Clinical Emergency County Hospital, 25 București Road, 500326, Brașov, Romania
[42] Anaesthesiology and Intensive Care Department, Reșița Emergency County Hospital, 8 Herculane Alley, 320154, Reșița, Romania
[43] Anaesthesiology and Intensive Care Department, Emergency County Hospital Dr. Fogolyán Kristóf, Stadionului Street, 520050, Sfântu Gheorghe, Romania
[44] Anaesthesiology and Intensive Care Department, Târgoviște Emergency County Hospital, 48 Tudor-Vladimirescu Street, 130002, Târgoviște, Romania
[45] Anaesthesiology and Intensive Care Department, Clinical Hospital of Pneumology and Infectious Diseases Dr. Victor Babeș, 126 București Road, 200515, Craiova, Romania
[46] Anaesthesiology and Intensive Care Department, Craiova Emergency County Hospital, 1 Tabaci Street, 200642, Craiova, Romania
[47] Department of Public Health, Babes-Bolyai University of Medecine and Pharmacy, 1 Mihail Kogalniceanu Street, Cluj, Romania
[48] General Surgery Department, Carol Davila University of Medicine and Pharmacy, 8 Eroii Sanitari Blvd., 050474, Bucharest, Romania
[49] Anaesthesiology and Intensive Care Department, Clinical Hospital of Obstetrics and Gynaecology Buna Vestire, 99 Nicolae Alexandrescu Street, 800151, Galați, Romania
[50] Anaesthesiology and Intensive Care Department,Clinical Emergency Hospital St. Apostol Andrei, 177 Brăilei Street, 800578, Galați, Romania
[51] Anaesthesiology and Intensive Care Department, Tecuci Municipal Hospital, 35 Costache Conachi Street, 805300, Tecuci, Romania
[52] Anaesthesiology and Intensive Care Department, Bolintin Vale City Hospital, 5 Republicii Street, 085100, Bolintin-vale, Romania
[53] Anaesthesiology and Intensive Care Department, Gheorgheni Municipal Hospital, 16 Lacu Roșu Blvd, 535500, Gheorgheni, Romania
[54] Anaesthesiology and Intensive Care Department, Odorheiu Secuiesc Municipal Hospital, 72 Bethlen Gabor Street, 535600, Odorheiu Secuiescu, Romania
[55] Anaesthesiology and Intensive Care Department, Petroșani Emergency Hospital, 137A, 1 Decembrie 1917 Street, 332058, Petroșani, Romania
[56] Anaesthesiology and Intensive Care Department, Deva Emergency County Hospital, 58 22 Decembrie Blvd. 330084, Deva, Romania
[57] Anaesthesiology and Intensive Care Department, Lupeni Municipal Hospital, Tudor Vladimirescu Street, 335600, Lupeni, Romania
[58] Anaesthesiology and Intensive Care Department, Clinical Hospital of Obstetrics and Gynaecology Cuza Vodă, 34 Cuza Vodă Street, 700038, Iași, Romania
[59] Anaesthesiology and Intensive Care Department, Clinical Hospital Dr. C. I. Parhon, 50 Carol I Blvd, 700503, Iași, Romania
[60] Anaesthesiology and Intensive Care Department, Institute for Cardiovascular Diseases Prof. Dr. George I.M. Georgescu, 50 Carol I Blvd. 700503, Iași, Romania
[61] Anaesthesiology and Intensive Care Department, Clinical Hospital of Obstetrics and Gynaecology Elena Doamna, 49 Elena Doamna Street, 700398, Iași, Romania

[62] Anaesthesiology and Intensive Care Department, Clinical Hospital for Infectious Diseases St. Parascheva, 2 Octav Botez Street, 700116, Iași, Romania
[63] Anaesthesiology and Intensive Care Department, Clinical Pneumology Hospital, 30 Dr. I. Cihac Street, 700115, Iași, Romania
[64] Anaesthesiology and Intensive Care Department, Emergency Clinical Hospital Prof. Dr. Nicolae Oblu, 2 Ateneului Street, 700309, Iași, Romania
[65] Anaesthesiology and Intensive Care Department, Emergency County Hospital St. Spiridon, 1 Independenței Blvd, 700111, Iași, Romania
[66] Anaesthesiology and Intensive Care Department, Pascani Municipal Hospital, 5 Gradiniței Street, 705204, Pascani, Romania
[67] Anaesthesiology and Intensive Care Department, Ilfov Emergency Clinical County Hospital, 49-51 Basarabia Blvd. 22104, Bucharest, Romania
[68] Anaesthesiology and Intensive Care Department, Hospital of Pneumology Dr. Nicolae Rușdea, 34 Dr. Victor Babeș Street, 430092, Baia Mare, Romania
[69] Anaesthesiology and Intensive Care Department, Drobeta Turnu-Severin Emergency County Hospital, 6D Mihai Viteazul Blvd. 220056, Drobeta Turnu-Severin, Romania
[70] Anaesthesiology and Intensive Care Department, Orșova Municipal Hospital, 34 Porțile de Fier Street, 225200, Orșova, Romania
[71] Anaesthesiology and Intensive Care Department, Emergency Institute for Cardiovascular Diseases and Transplant, 50 Gh. Marinescu Street, 540136, Târgu-Mureș, Romania
[72] Anaesthesiology and Intensive Care Departament, Târgu Mureș Clinical County Hospital, 1 Gheorghe Marinescu Street, 540136, Târgu Mureș, Romania
[73] Anaesthesiology and Intensive Care Departament, Piatra Neamț Emergency County Hospital, 1 Traian Blvd, 605200, Piatra Neamț, Romania
[74] Anaesthesiology and Intensive Care Departament, Ploiești Emergency County Hospital, 100 Găgeni Street, 100137, Ploiești, Romania
[75] Anaesthesiology and Intensive Care Departament, Câmpina Municipal Hospital, 117 Carol I Blvd., 105600, Câmpina, Romania
[76] Anaesthesiology and Intensive Care Departament, Zalău Emergency County Hospital, 67 Simion Bărnuțiu Street, 917151, Zalău, Romania
[77] Anaesthesiology and Intensive Care Departament, Carei Municipal Hospital, 25 octombrie Blvd., 445100, Carei, Romania
[78] Anaesthesiology and Intensive Care Departament, Sibiu Emergency County Hospital, 2-4 Corneliu Coposu Blvd., 550245, Sibiu, Romania
[79] Anaesthesiology and Intensive Care Departament, Suceava Emergency County Hospital, 21 1 Mai Blvd., 720224, Suceava, Romania
[80] Anaesthesiology and Intensive Care Departament, Institute for Cardiovascular and Heart Diseases of Timișoara, 13A Gheorghe Adam Street, 300310, Timișoara, Romania
[81] Anaesthesiology and Intensive Care Departament, Clinical Hospital of Infectious Diseases and Pneumophysiology Victor Babeș, 13 Gheorghe Adam Street, 300310, Timișoara, Romania
[82] Anaesthesiology and Intensive Care Departament, Timișoara Emergency Municipal Hospital, 5 Gheorghe Dima Street, 300254, Timișoara, Romania
[83] Anaesthesiology and Intensive Care Departament, Lugoj Municipal Hospital, 36 Gheorghe Doja Street, 305500, Lugoj, Romania
[84] Anaesthesiology and Intensive Care Departament, Military Emergency Hospital Dr. Victor Popescu, 7 Gheorghe Lazăr Street, 300080, Timișoara, Romania
[85] Anaesthesiology and Intensive Care Departament, Tulcea Emergency County Hospital, 32 1848 Street, 820180, Tulcea, Romania
[86] Anaesthesiology and Intensive Care Departament, Vaslui Emergency County Hospital, 233 Ștefan cel Mare Street, 730006, Vaslui, Romania
[87] Anaesthesiology and Intensive Care Departament, Bârlad Emergency Municipal Hospital, 300 Republicii Street, 731054, Bârlad, Romania

[88] Anaesthesiology and Intensive Care Departament, Adjud Municipal Hospital, 21 Republicii Blvd., 625100, Adjud, Romania
[89] Anaesthesiology and Intensive Care Departament, Clinical Hospital of Nephrology Dr. Carol Davila, 4 Griviței Road, 010731, Bucharest, Romania
[90] Anaesthesiology and Intensive Care Departament, Clinical Hospital for Ophthalmological Emergencies, 1 Alexandru Lahovari Plaza, 030167, Bucharest, Romania
[91] Anaesthesiology and Intensive Care Department, Clinical Institute for Urology, 4-6 Clinicilor Street, Cluj, Romania
[92] Anaesthesiology and Intensive Care Department, Tarnaveni Municipal Hospital, 2 Victor Babes Street, 545600, Tarnaveni, Romania
[93] Anaesthesiology and Intensive Care Department, George Emil Palade University of Medicine, Pharmacy, Science, and Technology, 38 Gh. Marinescu Street, 540142, Târgu Mureș, Romania
[94] Anaesthesiology and Intensive Care Department, Grigore T. Popa University of Medicine and Pharmacy, 16 Universității Street, 700115 Iași, Romania
[95] Anaesthesiology and Intensive Care Department, Regional Institute of Oncology, 2-4 Henri Berthelot Street, 700483, Iași, Romania
[96] Anaesthesiology and Intensive Care Department II, Iuliu Hațieganu University of Medicine and Pharmacy, 8 Victor Babeș Street, 400012, Cluj-Napoca, Romania
[97] Cardiovascular Surgery Department, Emergency Hospital, 8 Floreasca Road, 014461, Bucharest, Romania
[98] Cardiovascular Surgery Department, Carol Davila University of Medicine and Pharmacy, 8 Eroii Sanitari Blvd., 050474, Bucharest, Romania
[99] Anaesthesiology and Intensive Care Department, Emergency County Hospital Pius Brinzeu, 10 Iosif Bulbuca Blvd., Timișoara, Romania
[100] Anaesthesiology and Intensive Care Department, Victor Babeș University of Medicine and Pharmacy, 2 Eftimie Murgu Sq, 300041, Timișoara, Romania

Supplemental Table S1—Definitions.

Variable	Definition
Acute Respiratory Distress Syndrome	Berlin definition [48]
Acute Kidney Injury	KDIGO (Kidney Improving Global Outcomes) guidelines [49]
Major adverse cardiac events	Defined as one of the following:<br>- Non-fatal cardiac arrest. Absence of cardiac rhythm or the presence of chaotic rhythm needing any basic or advanced life support.<br>- Acute myocardial infarction.<br>- Newly identified congestive cardiac failure.<br>- New cardiac arrhythmia (atrial flutter or fibrillation) or block (grade II or III atrio-ventricular block) documented on ECG.<br>- Angor. Diffuse retrosternal discomfort induced by effort and ameliorated by rest or nitro-glycerine.
Myocardial infarction	Defined as an increase in serum levels of cardiac injury biomarkers (preferable, the troponin levels) > 99th percentile of the upper reference value associated with at least one of the following: cardiac ischemia symptoms, new or presumably new ST or T changings on ECG, or a left bundle branch block, pathological Q on ECG, imagistic or echocardiographic signs of a new loss in viable myocardium or alteration in segmentary kinetics, intracoronary artery identification of thrombi by angiography or autopsy [50]

Abbreviations: ECG—electrocardiography.

References:

[48] [ARDS Definition Task Force. Acute respiratory distress syndrome: the Berlin Definition. *JAMA*. 2012 Jun 20;307(23):2526-33.

[49] Levey AS, Eckardt KU, Dorman NM, Christiansen SL, Hoorn EJ, Ingelfinger JR, et al. Nomenclature for kidney function and disease: report of a Kidney Disease: Improving Global Outcomes (KDIGO) Consensus Conference. *Kidney Int*. 2020 Jun;97(6):1117-1129.

[50] Ibanez B, James S, Agewall S, Antunes MJ, Bucciarelli-Ducci C, Bueno H et al. 2017 ESC Guidelines for the management of acute myocardial infarction in patients presenting with ST-segment elevation: The Task Force for the management of acute myocardial infarction in patients presenting with ST-segment elevation of the European Society of Cardiology (ESC). *Eur Heart J.* 2018 Jan 7;39(2):119-177

Supplemental Table S2—Factorial analysis (2 factors).

Variables	Factor 1	Factor 2	Factor 3
Gender (male)		0.102	
SpO2 < 90% (yes)	0.160	0.548	-0.575
Dyspnoea (yes)		0.246	0.280
Mechanical Ventilation (yes)	0.842	0.129	0.128
HFNO2 (yes)	-0.627		
GCS (per 1 scale point)	-0.487	-0.154	
SOFA (per 1 scale point)	0.323	0.317	0.183
Corticotherapy (yes)		0.244	0.276

GCS—Glasgow Coma Scale; SOFA—Sequential organ failure assessment score; HFNO2—high flow nasal oxygen (meaning HFNO2 only, or the combination with non-invasive ventilation).

The hypothesis that two factors are sufficient was tested ($p<0.001$).

Supplemental Table S3—Propensity matching

	**Unmatched cohort**			**Matched cohort**		
**Variables**	Less elderly, n=4878	Extremely elderly, n=1666	SMD	Less elderly, n=1578	Extremely elderly, n=1578	SMD
Sex (male), n (%)	2811 (57.6)	834 (50.1)	0.152	827 (52.4)	808 (51.2)	0.024
Clinical status on ICU admission						
Fever (yes), n (%)	2016 (41.3)	561 (33.7)	0.159	532 (33.7)	548 (34.7)	0.021
Dyspnoea (yes), n (%)	3751 (76.9)	1288 (77.3)	0.01	1230 (77.9)	1223 (77.5)	0.011
SpO2 (%), mean ± SD	82.9 ± 9.8	83.2 ± 9.8	0.031	83.2 ± 10	83.1 ± 10	0.009
ARDS (yes), n (%)	1917 (39.3)	595 (35.7)	0.074	563 (35.7)	567 (35.9)	0.005
GCS, mean ± SD	12 ± 5	11 ± 5	0.222	11 ± 5	11 ± 5	0.021
SOFA, mean ± SD	9 ± 6	10 ± 6	0.134	9.4 ± 6	9.3 ± 6	0.025
Past medical history						
Ischemic heart disease (yes), n (%)	2059 (42.2)	972 (58.3)	0.327	913 (57.9)	888 (56.3)	0.032
Autoimmune disease (yes), n (%)	124 (2.5)	20 (1.2)	0.099	23 (1.5)	19 (1.2)	0.022
Chronic kidney disease (yes), n (%)	961 (19.7)	448 (26.9)	0.171	414 (26.2)	396 (25.1)	0.026
Dialysis patient (yes), n (%)	190 (3.9)	43 (2.6)	0.074	42 (2.7)	43 (2.7)	0.004
Diabetes type 1 (yes), n (%)	66 (1.4)	18 (1.1)	0.025	15 (1)	18 (1)	0.019
Diabetes type 2 (yes), n (%)	1841 (37.7)	509 (30.6)	0.152	525 (33.3)	491 (31.1)	0.046
Heart failure (yes), n (%)	1390 (28.5)	764 (45.9)	0.365	678 (43)	686 (43.5)	0.01
Arterial hypertension (yes), n (%)	3777 (77.4)	1339 (80.4)	0.072	1277 (80.9)	1269 (80.4)	0.013
COPD (yes), n (%)	495 (10.1)	156 (9.4)	0.026	162 (10.3)	153 (9.7)	0.019
Neoplasia (yes), n (%)	501 (10.3)	151 (9.1)	0.041	152 (9.6)	147 (9.3)	0.011
Patient management						
NIV (yes), n (%)	2415 (49.5)	729 (43.8)	0.115	694 (44)	704 (44.6)	0.013
MV (yes), n (%)	2584 (53)	886 (53.2)	0.004	844 (53.5)	828 (52.5)	0.020
Neuromuscular blockade (yes), n (%)	715 (14.7)	162 (9.7)	0.151	166 (10.5)	156 (9.9)	0.021
Prone position (yes), n (%)	763 (15.6)	175 (10.5)	0.153	177 (11.2)	168 (10.6)	0.018
Corticotherapy (yes), n (%)	4016 (82.3)	1295 (77.7)	0.115	1226 (77.7)	1269 (78.5)	0.018
Hydroxychloroquine (yes), n (%)	1085 (22.2)	356 (21.4)	0.021	350 (22.2)	338 (21.4)	0.018
Remdesivir (yes), n (%)	1290 (26.4)	316 (19)	0.179	297 (18.8)	309 (19.6)	0.019
Tocilizumab (yes), n (%)	693 (14.2)	138 (8.3)	0.188	146 (9.3)	136 (8.6)	0.022

ARDS—acute respiratory distress syndrome; COPD—chronic obstructive pulmonary disease; GCS—Glasgow Coma Scale; ICU—intensive care unit; SOF— sequential organ failure assessment.

Supplemental Figure S1. The principal component analysis considering the factors independently associated with ICU mortality in elderly patients.

MV—mechanical ventilation; HFNO2—high-flow nasal oxygen (corresponding to HFNO2 only, or HFO2 in combination with non-invasive ventilation); GCS—Glasgow Coma Scale; SOFA—sequential organ failure assessment; SpO2bin—SpO2 < 90% (yes); Gender—Male sex (yes).

The limit of less than 10 variables in the model was respected.

Suggestions for interpretation.

The represented variables explain 27+15 = 42% of the total variance. The GCS and HFNO2 are associated with survival and are diagonally positioned, comparing with MV and SOFA, so it should be considered as only one group of variables.

The SpO2<90%, dyspnoea, and corticotherapy variables form a second group of variables. The presence of one variable in one group might suggest a connection with the other variables of the group and speculates a (partially) common mechanism.

We note that the gender is very closed to the centre, meaning that it has a less accurate representation and a less significant role.

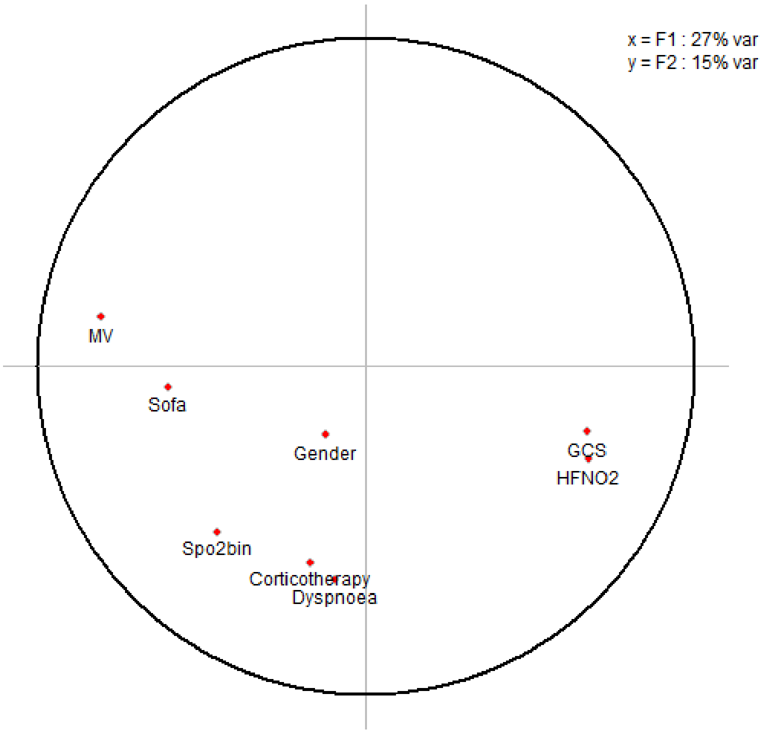

Supplemental Figure S2. The spherical principal component analysis considering the factors independently associated with ICU mortality in elderly patients.

Suggestions for interpretation.

This figure is a three-dimensional representation of Supplemental Figure 1. We note the diagonal positioning of HFNO2 and GCS comparing with SOFA and MV, but it should be considered as one group. The other group seems to be represented by SpO2, dyspnoea, and corticotherapy.

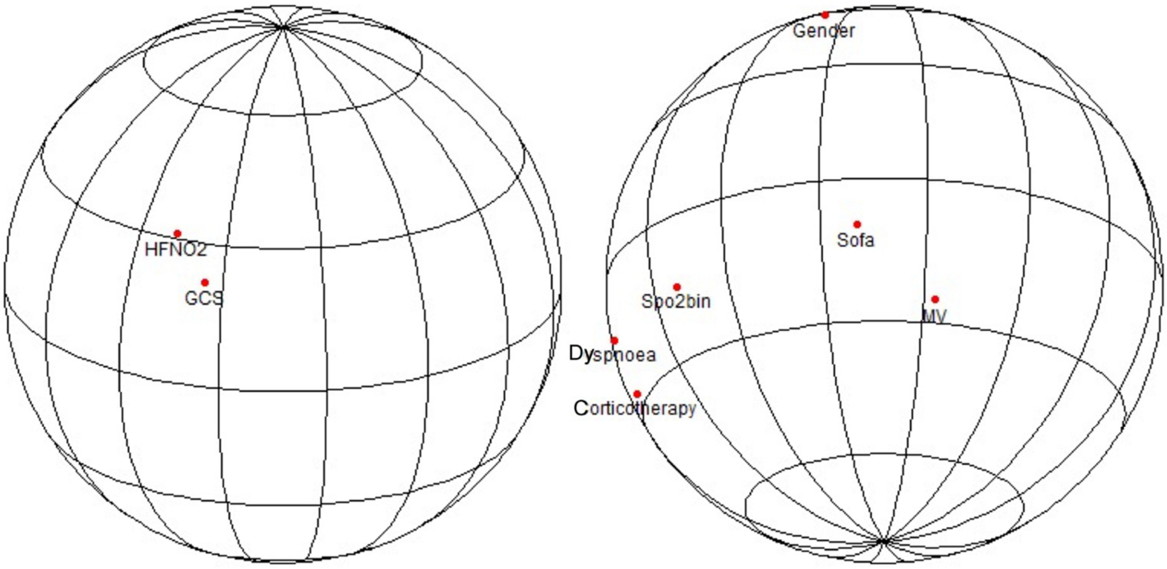

Supplemental Figure S3. The eigenvalues for factorial analysis considering the factors independently associated with ICU mortality in elderly patients.

Suggestion for interpretation

Two possible explaining factors are observed, as two points are over the noise-simulation (20 simulations) line.

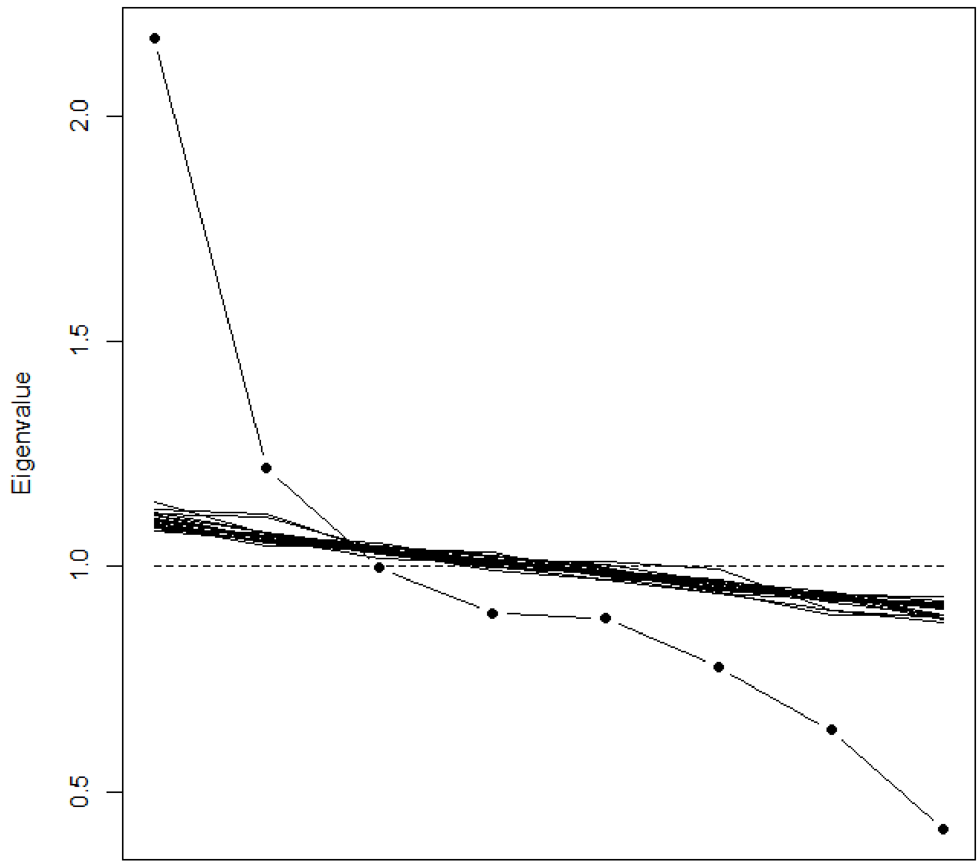

Supplemental Figure S4. The standardized mean difference (SMD) before and after matching.

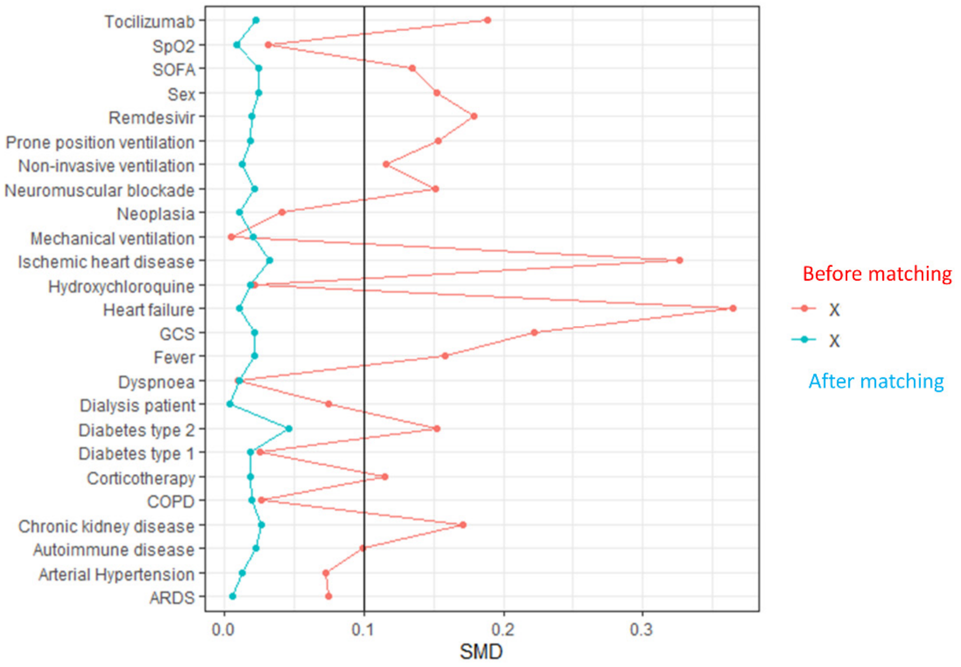

STROBE Statement—checklist of items that should be included in reports of observational studies.

	Item No.	Recommendation	Page No.
**Title and abstract**	1	(*a*) Indicate the study's design with a commonly used term in the title or the abstract.	1
		(*b*) Provide in the abstract an informative and balanced summary of what was done and what was found.	1
**Introduction**			
Background/rationale	2	Explain the scientific background and rationale for the investigation being reported.	2
Objectives	3	State specific objectives, including any prespecified hypotheses.	2
**Methods**			
Study design	4	Present key elements of study design early in the paper.	2
Setting	5	Describe the setting, locations, and relevant dates, including periods of recruitment, exposure, follow-up, and data collection.	2
Participants	6	(*a*) *Cohort study*—Give the eligibility criteria, and the sources and methods of selection of participants. Describe methods of follow-up.	2
		(*b*) *Cohort study*—For matched studies, give matching criteria and number of exposed and unexposed.	3
Variables	7	Clearly define all outcomes, exposures, predictors, potential confounders, and effect modifiers. Give diagnostic criteria, if applicable.	2-3
Data sources/ measurement	8	For each variable of interest, give sources of data and details of methods of assessment (measurement). Describe comparability of assessment methods if there is more than one group.	*3*
Bias	9	Describe any efforts to address potential sources of bias.	3
Study size	10	Explain how the study size was arrived at.	3

Continued on next page

Quantitative variables	11	Explain how quantitative variables were handled in the analyses. If applicable, describe which groupings were chosen and why.	3
Statistical methods	12	(*a*) Describe all statistical methods, including those used to control for confounding.	3
		(*b*) Describe any methods used to examine subgroups and interactions.	3
		(*c*) Explain how missing data were addressed.	3
		(*d*) *Cohort study*—If applicable, explain how loss to follow-up was addressed.	Not applicable
		(*e*) Describe any sensitivity analyses.	Not applicable
Participants	13	(a) Report numbers of individuals at each stage of study, e.g., numbers potentially eligible, examined for eligibility, confirmed eligible, included in the study, completing follow-up, and analysed.	4
		(b) Give reasons for non-participation at each stage.	4
		(c) Consider use of a flow diagram.	9
Descriptive data	14	(a) Give characteristics of study participants (e.g., demographic, clinical, social) and information on exposures and potential confounders.	4
		(b) Indicate number of participants with missing data for each variable of interest.	4
		(c) *Cohort study*—Summarise follow-up time (e.g., average and total amount)	Not applicable
Outcome data	15	*Cohort study*—Report numbers of outcome events or summary measures over time.	*4-8*
Main results	16	(*a*) Give unadjusted estimates and, if applicable, confounder-adjusted estimates and their precision (e.g., 95% confidence interval). Make clear which confounders were adjusted for and why they were included.	4-8
		(*b*) Report category boundaries when continuous variables were categorized.	4-8
		(*c*) If relevant, consider translating estimates of relative risk into absolute risk for a meaningful time period.	Not applicable

Continued on next page

Other analyses	17	Report other analyses done, e.g., analyses of subgroups and interactions, and sensitivity analyses.	5-6
Key results	18	Summarise key results with reference to study objectives.	10-11
Limitations	19	Discuss limitations of the study, taking into account sources of potential bias or imprecision. Discuss both direction and magnitude of any potential bias.	11-12
Interpretation	20	Give a cautious overall interpretation of results considering objectives, limitations, multiplicity of analyses, results from similar studies, and other relevant evidence.	11
Generalisability	21	Discuss the generalisability (external validity) of the study results.	11-12
**Other information**			
Funding	22	Give the source of funding and the role of the funders for the present study and, if applicable, for the original study on which the present article is based.	12
